# Integrating rapid exome sequencing into NICU clinical care after a pilot research study

**DOI:** 10.1038/s41525-022-00326-9

**Published:** 2022-09-05

**Authors:** Alissa M. D’Gama, Maya C. Del Rosario, Mairead A. Bresnahan, Timothy W. Yu, Monica H. Wojcik, Pankaj B. Agrawal

**Affiliations:** 1 Neonatal Genomics Program, Division of Newborn Medicine, Boston Children’s Hospital, Boston, MA USA; 2 Epilepsy Genetics Program, Division of Epilepsy and Neurophysiology, Department of Neurology, Boston Children’s Hospital, Boston, MA USA; 3 Division of Genetics and Genomics, Boston Children’s Hospital, Boston, MA USA; 4 Department of Pediatrics, Harvard Medical School, Boston, MA USA; 5 Broad Institute of MIT and Harvard, Cambridge, MA USA; 6 The Manton Center for Orphan Disease Research, Boston Children’s Hospital, Boston, MA USA

**Keywords:** Molecular medicine, Genetic testing, Genetics research, Genetic testing, Genetic testing

## Abstract

Genomic sequencing is a powerful diagnostic tool in critically ill infants, but performing exome or genome sequencing (ES/GS) in the context of a research study is different from implementing these tests clinically. We investigated the integration of rapid ES into routine clinical care after a pilot research study in a Level IV Neonatal Intensive Care Unit (NICU). We performed a retrospective cohort analysis of infants admitted with suspected genetic disorders to the NICU from December 1, 2018 to March 31, 2021 and compared results to those obtained from a previous research study cohort (March 1, 2017 to November 30, 2018). Clinical rapid ES was performed in 80/230 infants (35%) with a suspected genetic disorder and identified a genetic diagnosis in 22/80 infants (28%). The majority of diagnoses acutely impacted clinical management (14/22 (64%)). Compared to the previous research study, clinically integrated rapid ES had a significantly lower diagnostic yield and increased time from NICU admission and genetics consult to ES report, but identified four genetic diagnoses that may have been missed by the research study selection criteria. Compared to other genetic tests, rapid ES had similar or higher diagnostic yield and similar or decreased time to result. Overall, rapid ES was utilized in the NICU after the pilot research study, often as the first-tier sequencing test, and could identify the majority of disease-causing variants, shorten the diagnostic odyssey, and impact clinical care. Based on our experience, we have identified strategies to optimize the clinical implementation of rapid ES in the NICU.

## Introduction

Many infants admitted to Neonatal Intensive Care Units (NICUs) have a known or suspected genetic disorder, and these conditions contribute significantly to morbidity and mortality^1,2^. Traditionally, the genetic diagnostic odyssey has been long, costly, potentially invasive, and difficult for these infants and their families. Recently, there has been increasing use of massively-parallel sequencing tests, namely exome sequencing (ES) and genome sequencing (GS), for infants in intensive care settings. Multiple studies of ES/GS in critically ill infants have demonstrated diagnostic yields of 20–60% (with the highest yields reported with phenotype-driven selection), decreased time to genetic diagnosis (especially when rapid testing is performed), an impact on clinical care, and perception of high utility by clinicians and parents^3–20^.

We previously performed a prospective pilot study of rapid ES in critically ill infants using phenotype-based selection criteria, enrolling infants <6 months corrected gestational age (GA) with an ICU indication and new onset (<7 days) of hypotonia, seizures, a complex metabolic phenotype, and/or multiple congenital anomalies, as well as one infant with a disorder of sex development^11^. Between March 2017 and November 2018 (hereafter referred to as Phase I), 50 infants had rapid ES performed, mainly from the Level IV NICU at Boston Children’s Hospital (BCH). A genetic diagnosis was identified in 29/50 infants (58%), including two infants with variants identified in novel disease genes, and the highest yield was seen in infants with neurological phenotypes, specifically hypotonia and/or seizures. The diagnoses informed management in 24/29 infants (83%).

Subsequently, rapid ES was integrated into routine clinical care in our institution’s NICU. Unlike the research study in which a dedicated research team was readily available to identify and screen infants based on specific inclusion criteria, enroll and consent families, and collect and send samples, routine clinical care involves constantly rotating clinical team members with differing perspectives as well as competing clinical demands^21,22^. We therefore hypothesized that integration of rapid ES into routine clinical care may result in a reduction in rapid ES usage, varied phenotypic criteria applied to patient selection, and an increased time to report compared to rapid ES as part of a research study. We thus investigated our initial clinical implementation of rapid ES and its impact on the genetic diagnostic odyssey for infants in our institution’s NICU in the years following the completion of the pilot rapid ES research study.

## Results

### Characteristics of the study population

**Fig. 1:**
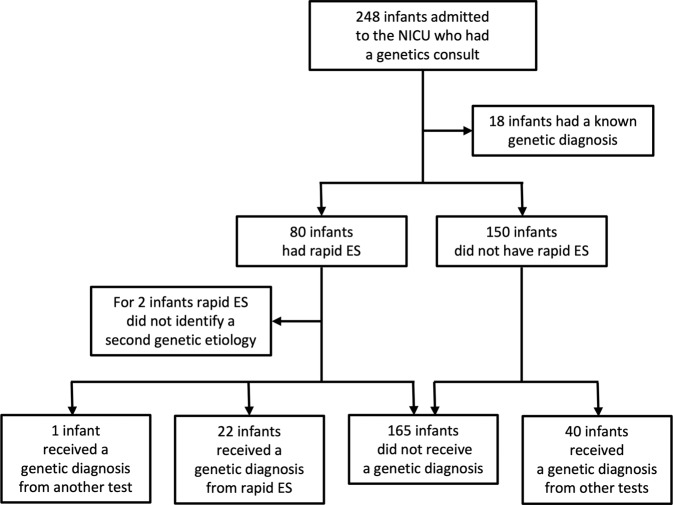
Genetic testing and genetic diagnoses in the NICU. Flowchart of the infants analyzed in Phase II and genetic diagnoses made.

During the December 2018 to March 2021 period (hereafter referred to as Phase II), 1230 infants were admitted to the Level IV NICU of our institution and 248 infants had a genetics consult. 18/248 infants (7%) had a known molecular genetic diagnosis (MGD) at the time of initial consult and were excluded from further analysis. Characteristics of the remaining 230 infants are summarized in Table 1 and the genetic diagnostic odyssey is summarized in Fig. 1. 130/230 infants were male (57%) and 100/230 were female (43%), with a median GA of 37 weeks (interquartile range (IQR) 34–39) and a median birth weight (BW) of 2665 grams (IQR 1980–3195). The most common reason for a genetics consult was one or more congenital anomalies (119/230 infants, 52%), followed by dysmorphic facial features (100/230 infants, 43%), neurologic phenotype (53/230 infants, 23%), and suspected metabolic disease (49/230 infants, 21%) (of note, each infant could have more than one reason for consult). A majority of the infants (168/230, 73%) required respiratory support, and 34/230 infants (15%) passed away in the first year of life.

**Table 1:** Demographics of infants admitted to the NICU in Phase II who had a genetics consult for an undiagnosed condition.

[number (%) unless otherwise noted]	Total *n* = 230	Got rapid ES *n* = 80	Did not get rapid ES *n* = 150	*p* value^a^
Male sex	130 (57)	41 (51)	89 (59)	0.265
GA (weeks; median (IQR))	37 (34, 39)	36 (33, 38)	37 (34, 39)	0.142
Prematurity <37 weeks	102 (44)	40 (50)	62 (41)	0.214
BW (grams; median (IQR))	2665 (1980, 3195)	2665 (1735, 3185)	2664.5 (2065, 3192.5)	0.417
Low BW <2500 grams	100 (43)	35 (44)	65 (43)	1
Age at genetics consult (days; median (IQR))	9 (3, 36)	10.5 (4, 52)	7 (3, 28)	0.108
Interval from NICU admission to genetics consult^b^ (days; median (IQR))	2 (1, 5)	2 (1, 4)	2 (1, 6)	0.985
*Phenotypic criteria*				
Neurologic (e.g., hypotonia, seizures)	53 (23)	31 (39)	22 (15)	<0.001
Congenital anomaly/anomalies	119 (52)	34 (43)	85 (57)	0.052
Suspected metabolic disease	49 (21)	21 (26)	28 (19)	0.236
Dysmorphic features	100 (43)	28 (35)	72 (48)	0.07
Failure to thrive	5 (2)	4 (5)	1 (1)	0.051
End of life	4 (2)	3 (4)	1 (1)	0.122
Family history of genetic disorder	6 (3)	1 (1)	5 (3)	0.667
Likely Mendelian disorder^c^	6 (3)	2 (3)	4 (2)	1
*Critical illness*				
Respiratory support (CPAP, NIPPV, or intubation)	168 (73)	65 (81)	103 (69)	0.0436
Inotropic support	65 (30)	28 (35)	37 (25)	0.124
Dialysis	9 (4)	4 (5)	5 (3)	0.723
Mortality (by 12 months)	34 (15)	21 (26)	13^d^ (9)	<0.001
Total BCH NICU Length of stay (days; median (IQR))	13 (5, 29)	21 (10, 46)	10 (3, 23.5)	<0.001

^a^Calculated using two-tailed Fisher’s exact test or Mann-Whitney test.

^b^For infants with initial genetics consult in our institution’s NICU.

^c^For example, disorder of sex development, interstitial lung disease, immunodeficiency.

^d^One additional infant passed away after one year.

### Rapid ES workflow: patient identification and eligibility

**Fig. 2:**
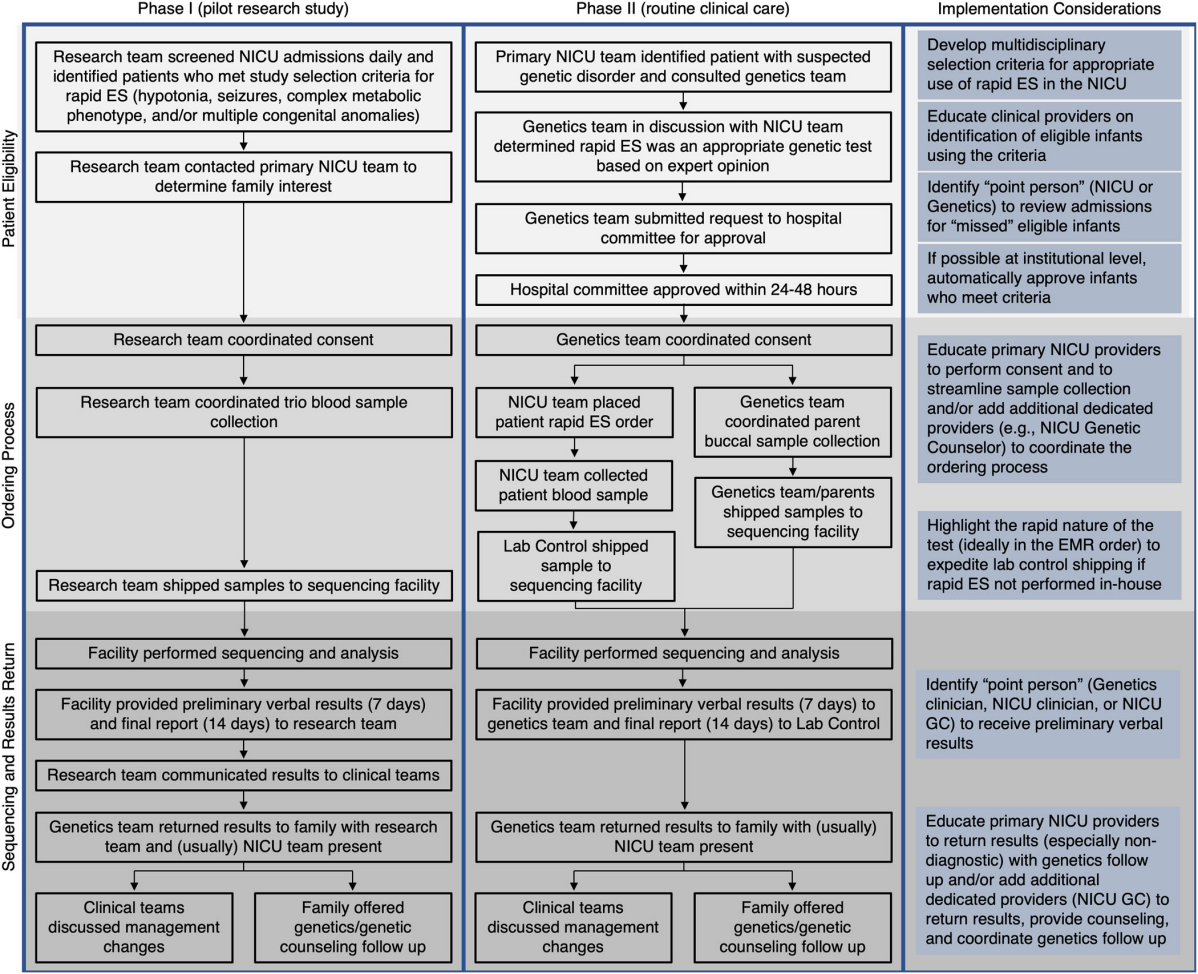
Comparison of the NICU rapid ES workflow in Phase 1 versus Phase II. Details of the rapid ES workflow in the pilot research study (Phase I) and subsequent integration into routine clinical care (Phase II) in the NICU. Potential strategies for optimizing implementation in routine NICU clinical care based on our experience are highlighted for each stage of the workflow.

The rapid ES workflow during the pilot research study (Phase I) compared to the rapid ES workflow after integration into routine clinical care in the NICU (Phase II) is summarized in Fig. 2. During Phase I, the research team screened new NICU admissions daily for infants who met the research study’s inclusion criteria and approached the family with the permission of the neonatology team. Although a genetics consult was not required for study enrollment, in practice the genetics team was always involved in clinical care.

During Phase II, the neonatology team identified infants suspected to have an underlying genetic disorder and consulted the genetics team. Although a genetics consult is not required to order rapid ES at our institution, in practice the genetics team was always involved and coordinated most of the rapid ES logistics. The geneticists, in discussion with the neonatologists, and if relevant, additional consulting teams like neurology, determined if rapid ES was an appropriate genetic test. We did not employ specific criteria for making this determination but instead depended on the expert opinion of the clinical teams on service. If rapid ES was determined to be an appropriate genetic test, a request form was submitted to a hospital committee made up of representatives from departments including genetics and laboratory medicine. In practice, the genetics team typically submitted this request and the committee decided to approve or deny the request within 24–48 hours.

During Phase II, a total of 80/230 infants (35%) with a genetics team consult for a suspected underlying genetic disorder had rapid ES performed. Baseline demographics were similar between infants who did versus did not have rapid ES performed. Age at genetics consult (median 10.5 days (IQR 4–52) versus median 7 days (IQR 3–28)) and time from NICU admission to genetics consult (for infants who had their initial genetics consult in our institution’s NICU; median 2 days (IQR 1–4) versus median 2 days (IQR 1–6)) were not significantly different between infants who did versus did not have rapid ES performed, respectively. Infants who had rapid ES performed were significantly more likely to have a neurologic phenotype (31/80 (39%) versus 22/150 (15%), *p* < 0.001) and to require respiratory support (65/80 (81%) versus 103/150 (69%), *p* = 0.044) compared to infants who did not have rapid ES performed. In addition, they had a significantly longer length of stay in our NICU (median 21 days (IQR 10–46) versus median 10 days (IQR 3–23.5), *p* < 0.001) and a significantly higher death rate in the first year of life (21/80 (26%) versus 13/150 (9%), *p* < 0.001). Age at genetics consult and time from NICU admission to genetics consult were not significantly different between infants who had rapid ES performed in Phase II versus Phase I (Table 2). Infants who had rapid ES performed in Phase I were significantly more likely to have a neurologic phenotype (28/35 (80%) versus 31/80 (39%), *p* < 0.001), congenital anomalies (26/35 (74%) versus 34/80 (43%), *p* = 0.002), and dysmorphic facial features (23/35 (66%) versus 28/80 (35%), *p* = 0.004) compared to infants who had rapid ES performed in Phase II.

**Table 2:** Comparison between Phase II and Phase I rapid ES in the NICU.

[days; median (IQR) unless otherwise noted]	Phase II *n* = 80^a^	Phase I *n* = 35	*p* value^b^
Diagnostic Yield (number (%))	22 (28%)	20 (57%)	0.003
Age at genetics consult	10.5 (4, 52)	7 (3, 26.5)	0.236
Interval from NICU admission to genetics consult^c^	2 (1, 4)	1 (1, 2)	0.114
Interval from genetics consult to sample collection	4 (2, 15.8)	3 (1, 7)	0.119
Interval from sample collection to ES report^d^	13 (10, 16.8)	13 (10, 14)	0.333
Interval from genetics consult to ES report	18 (15, 35)	16 (14, 19.5)	0.019
Interval from NICU admission to ES report	20 (16, 29)	17 (15, 19)	0.016
Age at ES report	45.5 (22, 98)	28 (18.5, 53)	0.015

^a^Two infants had ES sent at an outside hospital; dates not included in time intervals.

^b^Calculated using two-tailed Fisher’s exact test or Mann-Whitney test.

^c^For infants with initial genetics consult in our institution’s NICU.

^d^Date of ES report was abstracted as date of final (written) ES report.

### Rapid ES workflow: ordering process

During Phase I, the research team consented the family, coordinated the patient and parent blood sample collection, and shipped the trio samples directly to the sequencing facility. During Phase II, the genetics team (fellow and/or attending physician) consented the family, the primary neonatology team placed the rapid ES order in the electronic medical record (EMR for the patient blood sample collection, the genetics team coordinated parent buccal sample collection, our institution’s lab control shipped the patient sample to the sequencing facility, and the parents or genetics team shipped the parent samples directly to the sequencing facility. During both Phases, the consent process included the parents signing a consent form including whether they opted to receive secondary findings. The time from genetics consult to patient sample collection was not significantly different between Phase II and Phase I (median 4 days (IQR 2–15.8) versus median 3 days (IQR 1–7), *p* = 0.119).

### Rapid ES workflow: sequencing and results return

During both phases, the sequencing facility performed exome sequencing and analysis to detect single nucleotide variants (SNVs), small insertion/deletions (indels), and copy number variants (CNVs)^23^. Preliminary results were verbally returned within 7 days and a final report was returned within 14 days of receiving all samples (proband and parents where available). During Phase I, preliminary results and the final report were released to the research team, communicated by the research team to the clinical team, and the final report was scanned into the EMR. During Phase II, preliminary results were called to the provider specified in the order (generally a member of the genetics team), and the final report was released to our institution’s lab control and resulted as a lab in the EMR. Although the neonatology team could return results, in practice, results were returned to the family by the genetics team, often in the setting of a family meeting with the neonatology team also present. If the patient was discharged or had passed away, the genetics team contacted the family and returned results via phone call or clinic visit per family preference. Preliminary positive results were usually returned at the time of the verbal report with the disclaimer that the final report was still pending, and all results were retuned at the time of the final report. All families were offered a genetics clinic visit for further counseling and follow-up. The time from sample collection to final ES report was not significantly different between Phase II and Phase I (median 13 days (IQR 10–16.8) versus median 13 days (IQR 10–14), *p* = 0.333). The overall time from genetics consult to ES report was significantly longer in Phase II compared to Phase I (median 18 days (IQR 15–35) versus median 16 days (IQR 14–19.5), *p* = 0.019). The overall time from NICU admission to ES report was also significantly longer in Phase II compared to Phase I (median 20 days (IQR 16–29) versus median 17 days (IQR 15–19), *p* = 0.016).

### Diagnostic yield of rapid ES

A genetic diagnosis was made in 22/80 infants (28%) who had rapid ES in Phase II (Supplementary Table 1). Infants with diagnostic rapid ES had a significantly higher GA (median 37 weeks (IQR 36–39.8) versus median 36 weeks (IQR 32–37), *p* = 0.028) and BW (median 2945 grams (IQR 2270–3515) versus median 2600 grams (IQR 1550–3050), *p* = 0.011) compared to infants with non-diagnostic rapid ES (Table 3). The diagnostic yield of rapid ES in Phase II was significantly lower than the diagnostic yield of rapid ES in Phase I (22/80 (28%) versus 20/35 (57%), *p* = 0.003).

**Table 3:** Comparison between diagnostic and non-diagnostic rapid ES in the NICU in Phase II.

[number (%) unless otherwise noted]	Diagnostic *n* = 22	Non-diagnostic *n* = 58	*p* value^a^
Male sex	9 (41)	32 (55)	0.319
GA (weeks; median (IQR))	37 (36, 39.8)	36 (32, 37)	0.028
Prematurity <37 weeks	8 (36)	32 (55)	0.210
BW (grams; median (IQR))	2945 (2270, 3515)	2600 (1550, 3050)	0.011
Low BW <2500 grams	8 (36)	27 (47)	0.459
Age at genetics consult (days; median (range))	6 (3.3, 13)	15 (4, 61)	0.123
*Phenotypic criteria*			
Neurologic (e.g., hypotonia, seizures)	11 (50)	20 (34)	0.304
Congenital anomaly/anomalies	12 (55)	22 (38)	0.211
Suspected metabolic disease	4 (18)	17 (29)	0.401
Dysmorphic features	10 (45)	18 (31)	0.295
Failure to thrive	2 (9)	2 (3)	0.303
End of life	0 (0)	3 (5)	0.557
Family history of the genetic disorder	1 (5)	0 (0)	0.275
Likely Mendelian disorder	0 (0)	2 (3)	0.523
*Critical illness*			
Respiratory support (CPAP, NIPPV, or intubation)	17 (77)	48 (83)	0.749
Inotropic support	5 (23)	23 (40)	0.195
Dialysis	2 (9)	2 (3)	0.303
Mortality (by 12 months)	8 (36)	13 (22)	0.257

^a^Calculated using two-tailed Fisher’s exact test or Mann-Whitney test.

The pathogenic variants detected in Phase II were mostly SNVs or indels; exceptions included one infant with a homozygous partial gene deletion and one infant with multiple de novo CNVs. Of the cases with pathogenic SNVs or indels, eight were dominant de novo, eleven were autosomal recessive, and one was a maternally inherited X-linked condition. We observed a higher proportion of diagnoses involving recessive conditions (autosomal recessive or compound heterozygous) in Phase II compared to Phase I (55% versus 23%), though the significance of this is difficult to interpret due to cohort size. Identification of recessive conditions has particular importance for reproductive counseling for the families. The time from genetics consult to MGD was median 17.5 days (IQR 15–23.5) and the age at MGD was median 30 days (IQR 21–46.8) (exact date of diagnosis was not available for two infants with testing sent at an outside hospital). Rapid ES was the first genetic test sent based on our institution’s EMR for 16/22 infants diagnosed (73%). The genetic diagnosis was returned before discharge or death for 13/22 infants (59%), and 8/22 infants (36%) passed away in the first year of life. For two infants, rapid ES was sent due to concern for a second genetic etiology after a first genetic etiology had already been identified and did not identify an additional underlying genetic condition.

### Impact of rapid ES

To investigate the impact of the genetic diagnoses made by rapid ES on management, we focused on changes to management as described above in Materials and Methods. We did not include the impact on reproductive options as this was applicable to all cases and was consistently discussed by the genetics team with the family when returning results. We also did not include secondary findings, which included a diagnosis of G6PD deficiency in an infant that did not explain the infant’s presentation, and a pathogenic *BRCA2* variant identified in a parent. In total, genetic diagnoses impacted acute management in 14/22 infants (64%) (Supplementary Table 1). For three infants, the genetic diagnosis helped the family make the decision to transition to end-of-life care. For one of these infants, the decision to transition to end-of-life care was based on the preliminary verbal report and the infant had passed away at the time of the final report. For a fourth infant, the diagnosis was reported to provide reassurance to the family who had already transitioned to a modified “do-not-resuscitate order”; this case is not included in the 64% but highlights a psychosocial impact of genetic diagnosis. For two infants, the genetic diagnosis helped the family and medical team decide to pursue supportive care. For three infants, the genetic diagnosis led to a medication change, and for ten infants, to a new subspecialty evaluation.

### Utilization of rapid ES

**Fig. 3:**
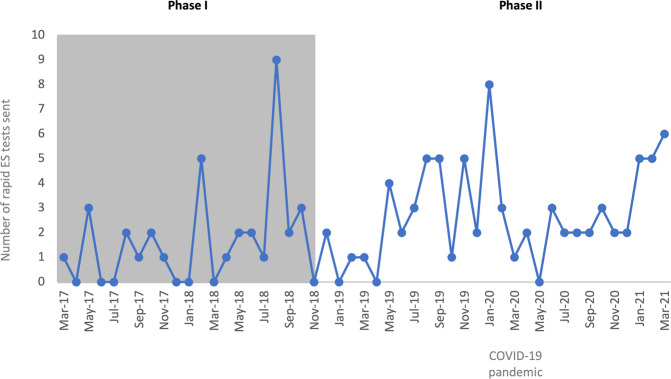
Utilization of rapid ES in the NICU. Number of rapid ES tests sent per month in our institution’s NICU during Phase I and Phase II.

Overall, rapid ES use decreased immediately after the Phase I study ended (Fig. 3), which likely reflected the initial transition period (Dec 2018—April 2019) from Phase I to Phase II. An increase in rapid ES utilization (May 2019—Jan 2020) then occurred until the start of the COVID-19 pandemic (Feb 2020—May 2020), when there was a decrease in utilization followed by gradual recovery in the number of rapid ES tests sent per month. We did not experience large decreases in the number of NICU admissions or number of genetics consults during this period, and the decrease likely reflected the transition of the genetics consult team to virtual only and institutional staffing shortages including in laboratory medicine during the peak of the COVID-19 pandemic. During Phase I an average of 1.7 rapid ES tests were sent per month, while during Phase II an average of 2.8 rapid ES tests were sent per month. Thus, although the COVID-19 pandemic temporarily disrupted utilization, clinicians in our NICU were utilizing rapid ES at a higher rate in routine clinical care within 6 months of completion of the research study.

### Genetic testing and diagnosis

Finally, we investigated the genetic tests sent and the genetic diagnoses made in the Phase II cohort overall (Supplementary Table 2). A total of 65 of 230 infants in the Phase II cohort received a genetic diagnosis (28%). In eight cases (four gene panel and four rapid ES), the genetic test detected variant(s) of uncertain significance (VUS) or combination of VUS and pathogenic (P)/likely pathogenic (LP) variants that the clinical genetics team considered MGD. There was a significant difference in yield between four types of genetic testing, with rapid ES yield 22/80 (28%), gene panel or single gene tests yield 24/96 (25%), karyotype, FISH, or CMA yield 13/129 (10%), and other genetic tests yield 6/68 (9%) (*p* < 0.001). Of note, other genetic tests included non-rapid ES (sent for 12 infants), which was ES sent as an outpatient that took 2–3 months to return. There was not a significant difference in the percentage of infants who received a genetic diagnosis prior to discharge or death between the four types of genetic testing. Time from consult to diagnosis was not significantly different when comparing infants who had to those who did not have rapid ES, but was significant when excluding those infants who had chromosome-level diagnoses (made by karyotype, FISH, or CMA) (Supplementary Fig. 1). There was a significant difference in time from consult to result between the four types of genetic testing; rapid ES and karyotype, FISH, or CMA had a significantly shorter time from consult to result compared to gene panel or single gene tests and other genetic tests (*p* < 0.001). These remained significant when removing infants who had non-rapid ES performed (*p* < 0.001 overall; *p* = 0.005 for rapid ES compared to other genetic tests).

There was only one case in which rapid ES did not identify a pathogenic variant that was identified on a different genetic test—an infant with congenital myotonic dystrophy for whom a myotonic syndrome gene panel identified the pathogenic expansion in *DMPK* (OMIM 160900). Two infants had a gene panel or single gene tests sent prior to ES which did not identify a genetic diagnosis that was subsequently identified on rapid ES—an infant diagnosed with pathogenic variants in *ALG12* (congenital disorder of glycosylation, OMIM 607143) who had negative *MPS1* gene testing and an infant with a pathogenic variant in *ADNP* (OMIM 615873) who had a negative Rubinstein-Taybi syndrome panel. Of the infants with pathogenic CNVs, only one infant had both rapid ES and CMA sent, and both tests identified the pathogenic CNVs.

Given that we collected data through September 2021 for infants admitted through March 2021 and there were thus some infants with less than 12 months of data, we conducted a sensitivity analysis for diagnostic yield and diagnosis prior to discharge or death restricting to genetic tests in the first six months of life, which did not change the statistical significance (there were only two infants who received a genetic diagnosis between six months and one year).

## Discussion

In this study, we describe the integration of rapid ES into routine clinical care in our Level IV NICU after the completion of a pilot research study. Overall, 248/1230 infants admitted to our institution’s NICU in Phase II had a genetics team consult, suggesting approximately one fifth of infants had a known or suspected underlying genetic disorder. Approximately one-third of infants with a genetics team consult in Phase II had rapid ES. These infants were more likely to have a neurological phenotype compared to those who did not have rapid ES. Although the infants were selected for rapid ES by the clinical team (neonatologist and consulting geneticist and/or neurologist) based on expert opinion and without specific selection criteria, this may reflect clinician recognition that neurological phenotypes have high yield on rapid ES, as previously demonstrated in Phase I^11^. These infants were also more likely to require respiratory support, which may reflect a tendency to prioritize rapid ES as a genetic test in more critically ill infants and may also be related to the high utilization among infants with neurological phenotypes (e.g., an infant with hypotonia dependent on positive pressure or an infant with seizures who required intubation and mechanical ventilation for airway protection). The increased length of stay and death within the first year for these infants likely reflects the known morbidity and mortality of genetic disorders that present in the neonatal period^2,17^.

Optimizing the rapid ES workflow is critical to the efficiency and sustainability of genomic sequencing in routine clinical care^21,22^. We found that the transition of rapid ES from the research study setting in Phase I into routine clinical care in Phase II was associated with a relatively lower diagnostic yield and an increased time from both NICU admission and genetics consult to ES report. We have identified several barriers to the implementation of rapid ES in routine clinical care, highlighted with potential strategies in Fig. 2 and discussed below. The Phase II yield of rapid ES was 28%, which is within the range reported for previous studies of ES/GS in NICUs and other ICU settings^3,4,6–20^. The lower diagnostic yield of Phase II compared to Phase I may reflect the shift from eligibility based on specific selection criteria in Phase I to eligibility based on the expert opinion of individual clinicians at the time of an infant’s NICU admission (which includes their knowledge and attitudes on genomic sequencing) and approval by the hospital oversight committee in Phase II. We screened the infants who received a diagnosis by rapid ES in Phase II based on the selection criteria used in Phase I, and found that 4/22 (18%) may not have qualified in Phase I (Supplementary Table 1). For example, a patient with Pierre Robin sequence and dysmorphic features was diagnosed with a pathogenic variant in *ADNP* (OMIM 615873) that led to new subspecialty evaluation. Thus, while rapid ES in Phase II had a decreased diagnostic yield compared to Phase I, it also identified genetic diagnoses in infants who may have had a longer or unresolved diagnostic odyssey in Phase I. We are currently implementing a quality improvement project in our NICU to establish optimized multidisciplinary selection criteria^24^ for rapid ES based on the Phase I and Phase II results and to provide education to neonatology providers on genomic medicine and practical use of these criteria. We acknowledge the need to balance the cost of rapid ES—which is currently bundled into the hospitalization cost at our institution such that the hospital bears the cost burden if insurance reimbursement for this service does not occur—with its ability to rapidly identify a broad range of genetic disorders. It is thus important for selection criteria to also specify when rapid ES is not appropriate—for example, for patients with features highly suggestive of a trisomy or other chromosomal anomaly, CMA is more appropriate as a first-line genetic test.

The longer time from NICU admission and genetics consult to ES report in Phase II compared to Phase I likely reflects the logistics of the rapid ES workflow described above now falling on providers from various teams with competing clinical demands compared to a dedicated, readily available research team. For critically ill infants, each day delay in diagnosis can matter for impact ranging from providing the family diagnostic closure to making decisions regarding goals of care. To understand barriers, we divided the rapid ES process into three major intervals: time from NICU admission to genetics consult, time from genetics consult to sample collection, and time from sample collection to ES report. The time from NICU admission to genetics consult is determined by the time it takes the primary NICU team to identify an admitted infant as having a suspected underlying genetic disorder and consult the genetics team. The time from genetics consult to sample collection is determined by 1) the time to determination of eligibility, 2) the time to consent the family, and 3) the time to place the order and collect trio samples. Determination of eligibility based on a research team performing daily screening using specific selection criteria is more efficient than the determination of eligibility based on the expert opinion of various clinicians and subsequent approval by a hospital committee. We are attempting to increase efficiency by optimizing selection criteria and providing provider education as discussed above, as well as by surveillance by our Neonatal Genomics Program. Consenting the family, which includes getting in contact with the family to arrange a time for consent and the actual consent process, currently falls on the genetics team at our institution. Placing the order and collecting trio samples is often delayed by confusion about specific order details for what is considered a special lab test and coordination of buccal swab sample collection from the parents. Education of primary neonatology providers (attendings, fellows, nurse practitioners, and nurses) who are at the bedside in real-time to consent and streamline sample collection and/or addition of a NICU Genetics Counselor who can focus on coordinating the ordering process are options we are currently exploring to improve efficiency.

The time from sample collection to final ES report is determined by 1) the time from sample collection to shipment to the sequencing facility and 2) the sequencing turnaround time at the sequencing facility. In Phase I, the trio samples were personally shipped to the sequencing facility by the research team immediately after collection. In Phase II, the patient sample was sent to lab control and shipped from our institution to the sequencing facility and the parent samples were sent by either the genetics team or the parents (using a prepaid shipping label and box) to the sequencing facility. Practically, it is not feasible for lab control at a large hospital to immediately ship every sample on arrival to the sequencing facility, although rapid genetic tests are expedited for approval and shipping. The turnaround time at the sequencing facility for sequencing and analysis was the same for both phases. Thus, our ongoing QI efforts are focused on the former intervals. Overall, we were encouraged that utilization did not decrease in Phase II and that the median time difference from genetics consult to ES report between the phases was only two days.

When comparing 1) rapid ES, 2) gene panel and single gene tests, 3) karyotype, FISH, and CMA, and 4) other genetic tests, several trends emerged. Traditionally, the tests in the second and third groups are the initial tests sent in a stepwise genetic evaluation, and ES may eventually be sent if these are non-diagnostic. On the one hand, rapid ES had a similar yield to gene panel and single gene tests (28% versus 25%, respectively), with a larger difference observed when we performed a sensitivity analysis removing cases where a metabolic diagnosis was known and genetic testing confirmed the diagnosis (26% vs 19%), but a shorter time to result. Of note, our data are likely an underestimate of the time to result for gene panel and single gene tests as the tests are sent only on the proband and thus parental phasing is often required to confirm a genetic diagnosis, and we were not always able to accurately determine the time interval to obtain subsequent phasing. Of the infants who had non-diagnostic gene panel or single gene tests, only two subsequently had rapid ES with diagnoses found and the majority remained undiagnosed at the end of the study period; thus it is unclear how many diagnoses may have been missed by this panel-first approach. Rapid ES had a higher yield than karyotype, FISH, and CMA (28% versus 10%), but a similar time to result.

Although we acknowledge that for certain classes of variants, such as short tandem repeat expansions, ES is currently suboptimal for detection, we suggest that rapid ES (or GS) can be an appropriate initial genetic test for the majority of NICU infants suspected to have an underlying genetic condition as a single test that optimizes both diagnostic yield and time to result. However, for infants with features highly suggestive of a trisomy or other chromosomal anomaly, CMA provides similarly rapid results at less cost. An infant with non-diagnostic ES at our institution has access to both reanalysis clinically and enrollment in a research study to perform reanalysis, GS, and/or functional studies, and thus there is the potential to identify a genetic diagnosis from the single ES test in the future as we continue to gain knowledge about disease genes and pathogenic variants^25^. Of the 55 infants with non-diagnostic rapid ES in Phase II, 15 (27%) are currently enrolled in the research study.

Important limitations of our study are the retrospective nature and decreased ability to fully capture the genetic diagnostic odyssey for infants who were not transferred to our NICU soon after birth (and may have had some genetic tests, especially cytogenetic tests, sent before transfer) or who were transferred from our institution to another institution where the genetic diagnosis was made after additional genetic testing. Our study is based on data from a NICU at a quaternary care referral center, which is likely enriched for admissions with congenital anomalies and underlying genetic disorders compared to a NICU at a birth hospital. In addition, we acknowledge the importance of understanding the cost implications of genomic sequencing and are including these measures in future QI efforts. However, our study provides a valuable description of the integration of rapid ES into routine clinical care in a NICU with a high volume of infants with underlying genetic conditions.

In conclusion, we find that integration of rapid ES into routine clinical care in the NICU after a pilot research study had a diagnostic yield within the range of previously published studies, rapid return of results and genetic diagnoses, and resulted in timely changes in clinical management in the majority of cases. Although clinically integrated rapid ES had a lower diagnostic yield and longer time to report than research study rapid ES, the diagnostic yield was still relatively high and it identified genetic diagnoses that would have been missed by the research study selection criteria. QI efforts are underway to optimize selection criteria and increase the efficiency of the rapid ES process. For clinicians working in the acute and busy NICU environment, we suggest that rapid ES represents a good choice for the majority of infants with suspected genetic disorders as an initial single genetic test (which generally requires only 1 ml of blood in a neonate) that captures the vast majority of currently known pathogenic genetic variants and shortens the difficult diagnostic odyssey for patients and their families, especially as the cost of genomic sequencing continues to decrease.

## Methods

### Consent

This retrospective study was approved by the Boston Children’s Hospital Institutional Review Board with a waiver of informed consent due to the nature of the study involving retrospective review of EMR. In both the pilot research study cohort and the clinical integration cohort, rapid ES was performed at GeneDx (Gaithersburg, MD). In the pilot research study cohort^11^, consent for rapid exome sequencing was performed by the research team and in the clinical integration cohort, consent for rapid exome sequencing was performed by the consulting genetics team.

### Data abstraction

We identified all infants admitted to the NICU of our institution between December 1, 2018 and March 31, 2021 who had a genetics team consult for an undiagnosed condition. Data related to the genetic evaluation and genetic diagnosis in the first year of life were abstracted from our EMR for each infant and stored in a Research Electronic Data Capture (REDCap) database hosted at our institution^26^. In addition to basic demographic data, we recorded the date, location, and reason for the initial genetics consult, the genetic testing recommended at the time of the initial consult, and the length of stay in the NICU or hospital; deceased status and date of death were also noted where applicable. Critical illness was defined by the need for respiratory support (CPAP, BiPAP/NIPPV, or intubation and mechanical ventilation), vasoactive medications, and/or dialysis. We recorded whether prenatal or other genetic testing was sent before the initial consult and collected details on those genetic tests when available. For each genetic test sent at BCH, we recorded the date collected, the date resulted, and whether the test resulted in a MGD. A MGD was defined when a P or LP variant(s) was identified on any genetic test that explained the infant’s presentation (identification of carrier status for an autosomal recessive condition was not counted as a genetic diagnosis). For each infant, we recorded whether a MGD was made in the first year of life, the testing modality leading to that diagnosis, and the date of diagnosis. For VUS, the clinical notes were reviewed to determine if these variants were considered clinically to represent a MGD. For infants with a MGD, we evaluated the impact on clinical management using the categories from the pilot research study: 1) transition to end-of-life/palliative care, 2) decision to pursue supportive care (tracheostomy and/or gastrostomy tube), 3) medication change or new therapeutic option, and/or 4) new subspecialty evaluation (including tests like echocardiography or magnetic resonance imaging). For the infants enrolled from the BCH NICU in the pilot research study, we reviewed the already collected data and the EMR to collect additional data on demographics and critical illness.

### Statistical analysis

Statistical analysis was performed using SPSS (Version 27.0, IBM Corp., Armonk, NY), using two-tailed Fisher’s exact, Mann-Whitney, or Kruskal-Wallis tests to compare variables as appropriate. Survival analysis was performed in R (Version 1.4.1717) using a Kaplan-Meier estimator and log rank test.

### Reporting summary

Further information on research design is available in the Nature Research Reporting Summary linked to this article.

## Supplementary information

Supplementary Dataset 1

Supplementary Information

Reporting Summary Checklist

## Data Availability

The genetic tests analyzed in this manuscript were ordered as clinical tests and thus the data is not able to be made available for privacy reasons. Rapid ES was performed by a CLIA-certified laboratory (GeneDx) as previously described^23^.
