# Supplementary material for: Integrating rapid exome sequencing into NICU clinical care after a pilot research study
Source: NPJ Genom Med. 2022 Sep 5;7:51. doi: 10.1038/s41525-022-00326-9 (PMC9441819; doi:10.1038/s41525-022-00326-9)
Supplement: Supplementary file 2 — Supplementary Information [file 41525_2022_326_MOESM2_ESM.pdf]

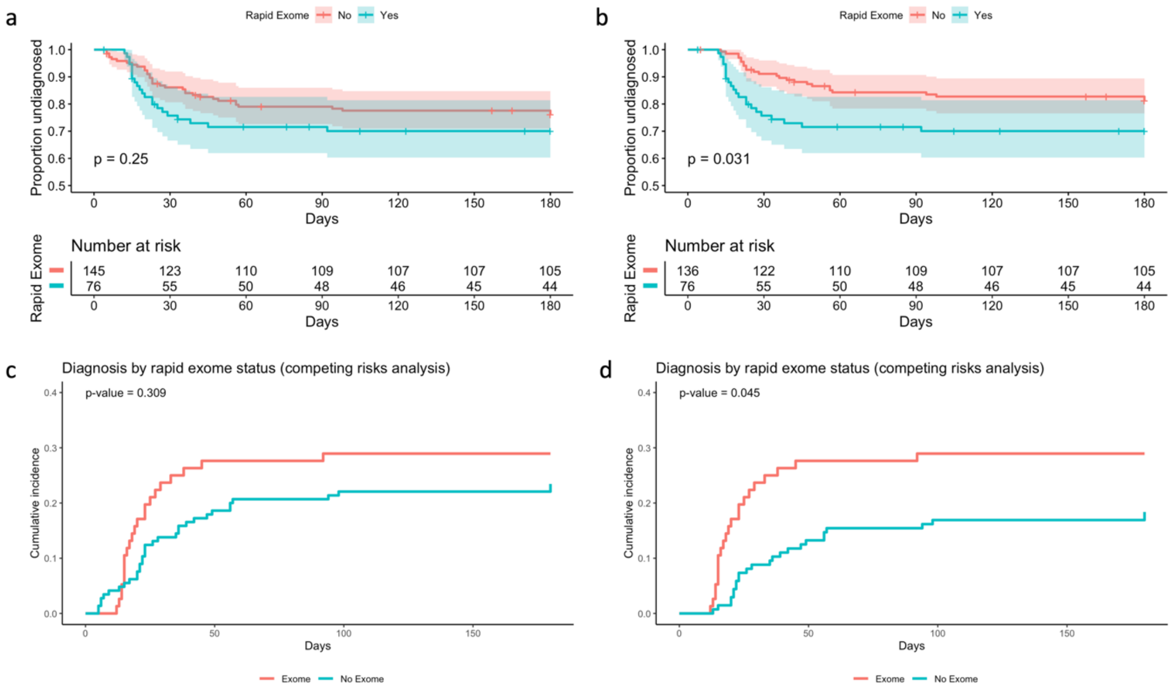

Supplementary Figure 1: Survival Analyses of Time to Diagnosis: a) Survival analysis of time to diagnosis, displaying proportion of infants remaining undiagnosed over the first 6 months following the initial genetics consult and comparing infants who had rapid ES (blue) to those who did not (red). Kaplan-Meier curves are presented including 95% confidence intervals (shading) with comparison by log-rank test. Risk set is displayed below the Kaplan-Meier plot. b) Survival analysis as in a) but excluding chromosomal-level diagnoses (made by karyotype, FISH, and chromosomal microarray analysis). c) Survival analysis of time to diagnosis over the first 6 months following the initial genetics consult incorporating the competing risk of death during this time period. Cumulative incidence of the event over time is presented, with comparison made by Gray's test (p = 0.308 for diagnosis, p = 0.066 for death). d) Survival analysis as in c) but excluding chromosomal-level diagnoses. Cumulative incidence of the event over time is presented, with comparison made by Gray's test (p = 0.045 for diagnosis, p = 0.094 for death).

Supplementary Table 2: Genetic testing and genetic diagnosis in the NICU in Phase II

	rapid exome sequencing n = 78 [80]	nonrapid exome sequencing n = 12	mito sequencing n = 29	gene panel n = 67 [73]	single gene test n = 21 [23]	CMA n = 82 [95]	karyotype n = 18 [24]	FISH n = 8 [10]	deletion/ duplication analysis n = 11	triplet analysis n = 2	methyl-ation analysis n = 11 [14]
Age at genetics consult (days; median, IQR)	11 (4, 54)	4 (2.8, 23)	11 (4, 44)	7 (2, 25)	11 (5, 44)	11 (4, 46.3)	2.5 (1.3, 18.8)	14.5 (3.3, 27.3)	10 (2.5, 31.5)	20.5 (10.8, 30.3)	12 (9.5, 22.5)
Interval from genetics consult to sample collection (days; median, IQR)	4 (2, 15.8)	176 (94.3, 247)	4 (2, 17)	5 (2, 23.3)	4 (1, 15)	2 (1, 6)	2.5 (1, 10.8)	7 (1, 32.3)	17 (3.5, 49)	102.5 (52.3, 152.8)	3.5 (1, 5.5)
Interval from sample collection to result (days; median, IQR)	13 (10, 16.8)	81 (54.3, 90)	21 (18, 29)	29 (22.5, 37.5)	29 (23, 34)	16 (7, 21)	6 (5.3, 9.3)	27 (13.5, 47.3)	20 (14, 36)	26.5 (24.3, 28.8)	27 (19, 41.5)
Interval from genetics consult to result (days; median, IQR)	18 (15, 35)	234.5 (171.3, 347.8)	24 (21, 51)	39 (27, 65)	35 (26, 57)	20 (10.3, 28)	8.5 (7, 17)	39.5 (16.5, 106)	42 (34, 86)	129 (76.5, 181.5)	34 (25, 46)
Age at result (days; median, IQR)	45.5 (22, 98)	287 (190.8, 372)	62 (37, 114)	59 (34.5, 102.5)	57 (38, 113)	35.5 (23, 78)	13.5 (9.3, 56.3)	97.5 (22, 144.5)	61 (40, 92)	149.5 (87.3, 211.8)	55 (40.5, 63)
Yield (genetic diagnoses made in the first year (%))	21 (27)	1 (8)	0 (0)	15 (22)	7 (33)	6 (7)	2 (11)	1 (13)	0 (0)	0 (0)	3 (27)
Yield by phenotypic criteria (%)											
Neurologic (e.g., hypotonia, seizures)	10/30 (33)	1/4 (25)	0	2/9 (22)	0/2 (0)	1/23 (4)	0/3 (0)	0/3 (0)	0/6 (0)	0	2/7 (29)
Congenital anomaly/anomalies	11/32 (34)	1/9 (11)	0	4/41 (10)	4/7 (57)	6/47 (13)	1/15 (7)	0/3 (0)	0/3 (0)	0/1 (0)	1/3 (33)
Suspected metabolic disease	4/21 (19)	0/1 (0)	0	8/13 (62)	2/9 (22)	0/12 (0)	0	0/1 (0)	0/1 (0)	0	0
Likely Mendelian disorder (e.g., DSD, ILD, ID)	0/2 (0)	0/1 (0)	0	1/2 (50)	0	0/1 (0)	0/1 (0)	0	0	0	0/1 (0)
Dysmorphic features	9/26 (35)	0/5 (0)	0	2/27 (7)	2/6 (33)	5/39 (13)	2/11 (18)	1/4 (25)	0/6 (0)	0/2 (0)	1/3 (33)
Failure to thrive	2/4 (50)	0	0	0	0	0/2 (0)	0	0	0/1 (0)	0	0
End of life	0/3 (0)	0	0	0/1 (0)	0	0	0	0	0	0	0
Family history of genetic disorder	1/1 (100)	0/2 (0)	0	0/3 (0)	1/1 (100)	0/2 (0)	0	0	0	0	0
For infants with diagnostic genetic testing											
Interval from genetics consult to genetic diagnosis (days; median, IQR)	17.5 (15, 23.5)	329	n/a	26 (22, 40.5)	35 (26.5, 46.5)	13 (7.5, 21.5)	5.5 (5.3, 5.8)	5	n/a	n/a	49 (34.5, 52.5)
Age at diagnosis (days; median, IQR)	30 (21, 46.8)	333	n/a	31 (26.5, 54.5)	49 (34.5, 62)	20.5 (10.3, 36)	7 (7, 7)	5	n/a	n/a	57 (46, 58.5)
Diagnosis prior to discharge/death (%)	12/21 (57)	0/1	n/a	7/15 (47)	2/7 (29)	4/6 (67)	1/2 (50)	1/1 (100)	n/a	n/a	0/3 (0)

Infants may have had more than one genetic test sent.
In the first row, n = number of tests sent at BCH [total number of tests sent].
Four infants who received gene panel sequencing and four infants who received rapid ES had VUS or a combination of P/LP and VUS detected that were considered molecular genetic diagnoses by the clinical genetics team.
